# Sintering Temperature-Dependence on Radiopacity of Bi_(2−x)_ ZrxO_(3+x/2)_ Powders Prepared by Sol-Gel Process

**DOI:** 10.3390/ma11091685

**Published:** 2018-09-11

**Authors:** May-Show Chen, Shih-Hsun Chen, Fu-Chih Lai, Chin-Yi Chen, Ming-Yuan Hsieh, Wei-Jen Chang, Jen-Chang Yang, Chung-Kwei Lin

**Affiliations:** 1 School of Oral Hygiene, College of Oral Medicine, Taipei Medical University, Taipei 11031, Taiwan; mayshowc@hotmail.com; 2 Department of Dentistry, Taipei Medical University Hospital, Taipei 11031, Taiwan; 3 Department of Mechanical Engineering, National Taiwan University of Science and Technology, Taipei 10607, Taiwan; shchen@mail.ntust.edu.tw; 4 College of Nursing, Taipei Medical University, Taipei 11031, Taiwan; flai@tmu.edu.tw; 5 Department of Materials Science and Engineering, Feng Chia University, Taichung 40724, Taiwan; chencyi@fcu.edu.tw (C.-Y.C.); shchen@northwestern.edu (M.-Y.H.); 6 School of Dentistry, College of Oral Medicine, Taipei Medical University, Taipei 110, Taiwan; cweijen1@tmu.edu.tw; 7 Dental Department, Taipei Medical University, Shuang-Ho Hospital, New Taipei 235, Taiwan; 8 Graduate Institute of Nanomedicine and Medical Engineering, College of Biomedical Engineering, Taipei Medical University, Taipei 110-52, Taiwan; 9 School of Dental Technology, College of Oral Medicine, Taipei Medical University, Taipei 11031, Taiwan; 10 Research Center of Digital Oral Science and Technology, College of Oral Medicine, Taipei Medical University, Taipei 11031, Taiwan

**Keywords:** mineral trioxide aggregate, radiopacity, bismuth oxide, zirconia, sol-gel process

## Abstract

Bismuth oxide (Bi_2_O_3_) is an effective additive used to enhance radiography resolution for dental materials. However, there are potential concerns regarding its biocompatibility and connection to tissue discoloration. In the present study, we modified the radiopacity properties of Bi_2_O_3_ with zirconium oxide (ZrO_2_) using a sol-gel process and investigated the composition, as well as the effects of heat treatment temperature using Thermogravimetry analysis (TGA), differential scanning calorimetry (DSC), Fourier transform infrared spectroscopy (FT-IR), transmission electron microscopy (TEM), and X-ray diffraction (XRD). The harvested Bi_2−x_Zr_x_O_3+x/2_ particles showed that the dominant phase transferred from *α*-Bi_2_O_3_ to *β*-Bi_7.38_Zr_0.62_O_12.31_ after a heat treatment of over 750 °C for 2 h. As the x values of Bi_2−x_Zr_x_O_3+x/2_ increased from 0.2 to 1.0, more zirconium oxide precipitated onto the particle surface, thus enhancing the surface roughness of particles. For sol-gel Bi_1.8_Zr_0.2_O_3.1_ powders (x = 0.2), the radiopacity values became 4.90 ± 0.23 and 5.83 ± 0.22 mmAl after a heat treatment of 500 °C and 750 °C, respectively.

## 1. Introduction

The proper level of radiopacity is of great importance for some dental materials to contrast them from intra-oral surrounding structures [1,2]. Dental materials such as root canal filling material [3], glass ionomer [4], cavity liners [5], luting cements [6], impression materials [7], restorative materials [8], and composite-resin materials [9] are usually loaded with radiopaque filler, typically dense metal or ceramic powders, to attenuate X-ray radiation energy for providing the visual contrast image of the device in the body. Depending on the intended use of dental materials, the minimum radiopacity requirement can vary. For polymer-based restorative materials recommended by ISO 4049/2009 [10], the radiopacity requirement is 1.0 mmAl of the material, while the radiopacity requirement for root canal sealing materials is above 3.0 mmAl, according to ISO 6876/2001 [11].

An ideal root canal filling material should present sufficient radiopacity to distinguish filling material from surrounding anatomical structures [12]. Mineral trioxide aggregate (MTA), a powder mixture comprising of hydrophilic particles of Portland cement (PC), clinker and bismuth oxide, is extensively used as a root canal sealer and for pulp capping, apexification, and root end filling [13,14,15,16]. Bismuth has a high atomic number (Z = 83) that allows it to reduce the amplitude of incident X-ray beam and create the root canal image inside the gums. For X-ray radiographic application, the radiopacity of Portland cement is about 0.86–2.02 mmAl, which is lower than the criteria of 3.0 mmAl [17]. However, the radiopacity can be significantly increased to 6–8 mmAl by adding 20% bismuth oxide [18,19,20,21].

Although there exists widespread clinical acceptance of MTA, many research findings regarding reduction in biocompatibility [22], tissue discoloration [23], and mechanical property [24] were attributed to the addition of bismuth oxide. Cavicchi et al. reported that the cytotoxicity of Bi_2_O_3_ containing PC was statistically higher at 12 and 24 h but later gradually decreased to the level of PC. Thus, efforts were devoted towards seeking a replacement for bismuth oxide [25,26,27]. There are various additives, but, currently, only gold and silver–tin alloy powders provide a sufficient radiopacity higher than dentin [28,29]. Thus, a more suitable MTA additive with a higher radiopacity and biocompatibility will be a valuable contribution for root canal treatment.

According to previous reports, the modified radiopacifier of Bi_2_O_3_ with yttria-stabilized zirconia (YSZ) via solid state reaction resulted in a higher radiopacity but similar cell viability when compared to pure Bi_2_O_3_ [27]. Unlike the conventional solid state reaction, sol-gel technology is a well-established colloidal chemistry technology, which offers the potential to produce high purity and uniform nanostructures and is achievable at low temperatures [30]. To suppress undesirable effects caused by bismuth oxide, many studies have been performed to find alternatives with both good biocompatibility and mechanical properties. Among them is zirconium oxide, a nearly-bioinert material that is usually used in oral cavities due to its high compatibility, mechanical strength, good abrasion resistance and chemical stability [31,32]. With their advantages of low production cost and easy operation, wet chemical processes are generally used for preparing oxide materials [33,34,35,36]. This study proposes that the introduction of zirconium ions to prepare the radiopacifier of Bi_2−x_Zr_x_O_3+x/2_ powders via sol-gel process can reduce intrinsic cytotoxicity. Furthermore, the effects of different zirconium additive ratios and heat-treatments were investigated, and the radiopacity of the obtained powders were also assayed.

## 2. Materials and Methods

In this study, Bi_2−x_Zr_x_O_3+x/2_ powders were prepared using the sol-gel process, and were further modified through controlling heat treatment conditions. All chemicals were of analytical grade and used as received from J.T. Baker, Japan without any further purification. Bismuth nitrate pentahydrate (Bi(NO_3_)_3_∙5H_2_O) and zirconyl nitrate hydrate (ZrO(NO_3_)_2_∙H_2_O) were used as precursors for Bi_2−x_Zr_x_O_3+x/2_ synthesis and the x value varied from 0 to 1.0, 0.2 each.

Ten grams of bismuth nitrate pentahydrate (Bi(NO_3_)_3_∙5H_2_O) were mixed with 10 mL of glacial acetic acid (CH_3_COOH) under mild stirring at 40 °C for 3 h. The resulting solution was introduced into a 100 mL vessel, and then 2-methoxyethanol (2-MOE, C_3_H_8_O_2_) and zirconyl nitrate hydrate (ZrO(NO_3_)_2_∙H_2_O) with various ratios were added. In a typical sol-gel process, 2-MOE was usually used to control pH, viscosity, liquid surface tension, and form a stable complex without particles sedimentation [37]. After stirring at 40 °C for 3 h, the mixture appeared transparent, and turned into gel state after another 48 h of stirring. The obtained products were dried in a vacuum oven at 90 °C overnight and ground into fine powders using a mortar and pestle for the following heat treatments at different temperatures. The MTA-like cements were prepared by mixing Portland cement/radiopacitifier using a benchtop planetary ball mill (Retsch PM100, Haan, Germany) for 10 min milling time. Each harvested cement was mixed at a powder-to-liquid (P/L) ratio of 0.4 g powder per 0.1 mL liquid, loaded into a Teflon ring (10 mm diameter with 1 mm thickness), and set at 37 °C for 24 h. Six specimens (N = 6) were prepared for radiopacity measurement.

The obtained dried powders were heat treated for 2 h at different temperatures, ranging from 500 to 800 °C. Thermogravimetry analysis (Perkin Elmer Pyrsis 1 TGA) and differential scanning calorimetry (DSC, Simultaneous SDT 2960, TA instruments Ltd., New Castle, DE, USA) were used to examine the thermal stability and transformation. Powder X-ray diffraction (XRD) patterns of the as-prepared samples were analyzed on a SRAM18XHF X-ray powder diffractometer (MacScience Co. Ltd., Tokyo, Japan). The binding energies were measured using Fourier transform infrared spectroscopy (FT-IR spectrometer frontier, Perkin Elmer). The morphologies and microstructure were observed using field emission scanning electron microscopy (FE-SEM, JSM-6700F) and transmission electron microscopy (TEM, JEOL-2100F) operated at an accelerating voltage of 200 kV. Furthermore, the radiopacity of the obtained powders was determined using a dental X-ray system (VX-65, VATECH ENG Co. Ltd., Gyeonggi-do, Korea) operated at 62 kV, 10 mA, 0.64 s exposure time, and 30 cm focus-film distance. The defect-free specimens were positioned on occlusal radiographic films (Koadak CR imaging plate size 2; Eastman-Kodak Co., Rochester, NY, USA) and exposed along with an aluminium step-wedge with variable thickness (from 2 to 16 mm in 2 mm increments). The mean gray values of each step of the aluminum step wedge and the specimens were measured by outlining a region of interest using the equal-density area tool of the imaging processing software, Image J 1.39f (Wayne Rasband, National Institutes of Health, Bethesda, MD, USA). Each data point and error were calculated by averaging 10 repeated measurements.

## 3. Results and Discussion

### 3.1. Characterization of As-Fabricated Bi_2_O_3_ Powders

The effects of the zirconium additive ratio were investigated in this study and the basic properties of the primitive bismuth oxide powders synthesized using the sol-gel process were first recorded and are shown below.

### 3.2. Thermal Properties

**Figure 1:**
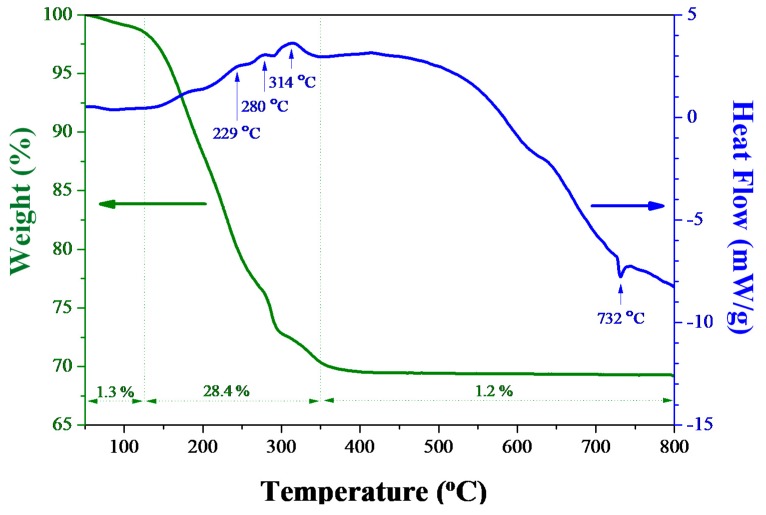
DSC/TGA analytic results of the as-synthesized sol-gel Bi_2_O_3_ powders.

Figure 1 shows the DSC/TGA analytic results of the as-synthesized sol-gel Bi_2_O_3_ powders. The measuring temperature ranged from 50 to 800 °C with a heating rate of 10 °C/min, while air flow was introduced to simulate the following heat-treatment conditions. The TGA curve of sol-gel Bi_2_O_3_ powder contains three steps: (i) 50–120 °C, evaporation of physical-adsorbed water on bismuth oxide powders, resulting in about 1.3% weight loss; (ii) 120–350 °C, thermal decomposition and burnout of glacial acetic acid and 2-MOE, resulting in 24.8% weight loss; and (iii) 600–800 °C, steady state. The total weight loss was 30.9% after a 50–800 °C heat treatment. DSC result further reported that three exothermic peaks occurred at 229, 282, and 314 °C, resulting from the decomposition and burnout of acetate, nitrite and organics [38,39,40]. As the temperature increased, another endothermic peak appeared at 732 °C due to the transformation of bismuth oxide from *α* to *δ* phase [41].

### 3.3. Crystallization and Microstructure

**Figure 2:**
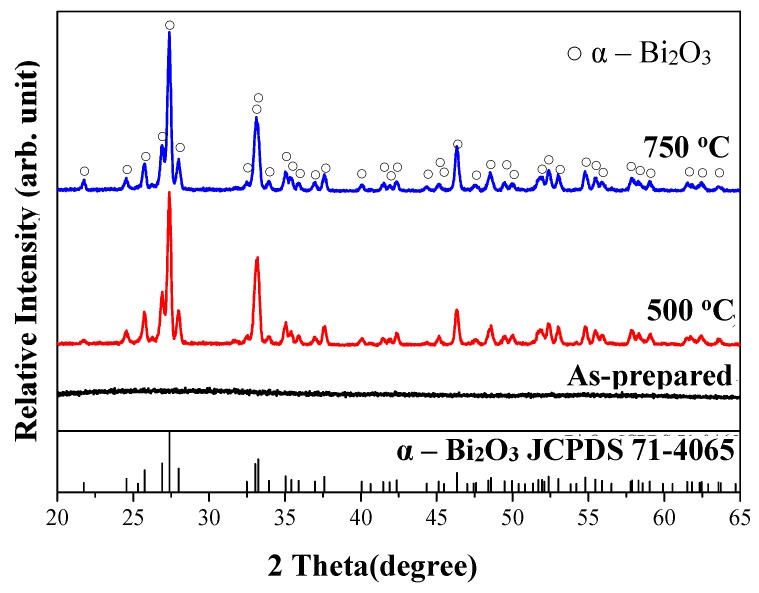
XRD patterns of as-obtained and heat-treated Bi_2_O_3_ powders prepared using the sol-gel process.

XRD patterns of Bi_2_O_3_ powders prepared using the sol-gel process are shown in Figure 2. The results indicate that the as-prepared Bi_2_O_3_ product was non-crystalline. After 2 h of heating to 500 °C at a rate of 10 °C/min, some characteristic sharp peaks were detected, corresponding to 26.9°, 27.4°, 28.0°, and 33.2°. The observed XRD peaks of Bi_2_O_3_ diffraction patterns can be attributed to monoclinic *α*-Bi_2_O_3,_ based on JCPDS card # 71-0465. After raising the temperature to 750 °C, no evident difference was further observed.

**Figure 3:**
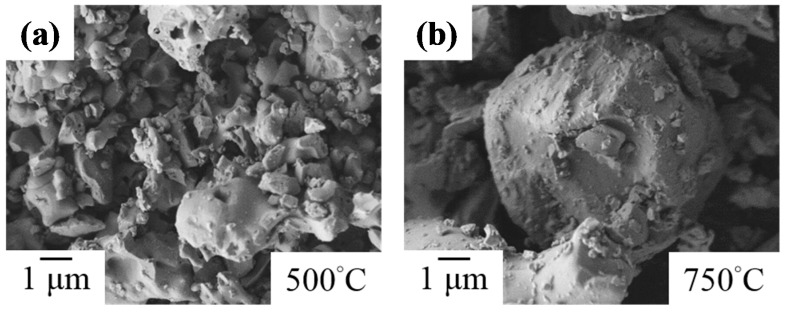
SEM images of sol-gel Bi_2_O_3_ powders after heat-treatment at: (**a**) 500 °C; and (**b**) 750 °C.

SEM images of heat-treated Bi_2_O_3_ powders are compared in Figure 3. Heat-treated products presented an irregular appearance at 500 °C and further aggregated in large particles after a heat treatment at 750 °C. It was identical to the phenomenon reported by Harwig et al., which stated that the high-temperature stable *δ*-Bi_2_O_3_ phase forms above 730 °C and undergoes a partial liquid phase sintering process [42].

**Figure 4:**
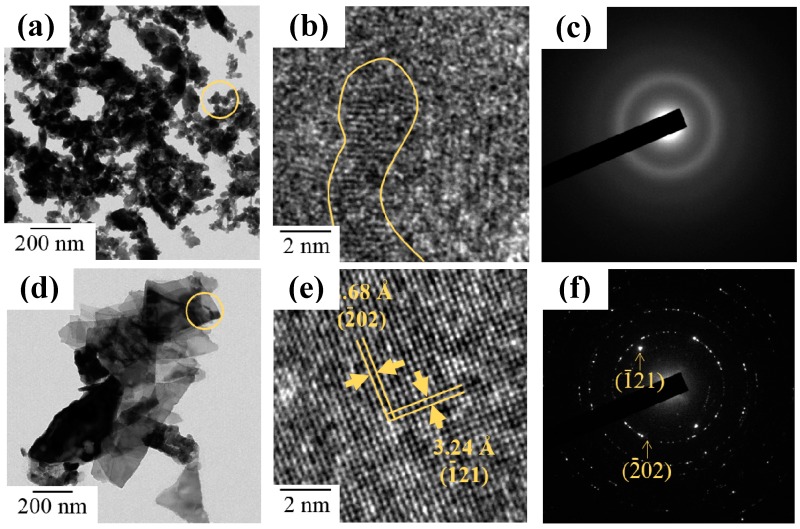
TEM images of sol-gel Bi_2_O_3_ powders (**a**) before and (**d**) after 500 °C heat treatment. (**b**,**e**) HRTEM images; and (**c**,**f**) selected area diffraction pattern (SAED) taken from the circled area in (**a**,**d**), respectively.

TEM observation was further used to investigate the heat effect on the microstructure of the sol-gel Bi_2_O_3_ powders. Figure 4a shows the TEM image of as-synthesized sol-gel Bi_2_O_3_ powders, which consisted of fine particles of irregular outline. Based on its high resolution TEM image (HRTEM) in Figure 4b, almost no periodical order could be observed, and its selected area diffraction pattern (SAED) in Figure 4c formed diffraction rings, suggesting the existence of nanocrystalline structure. After a heat treatment at 50 °C, Bi_2_O_3_ powders aggregated in larger lamellar particles and the inter-planar spacing of 3.24 and 2.68 Å are consistent with the d values and planes of monoclinic *α*-Bi_2_O_3_, as shown in Figure 4d,e. In addition, SAED patterns further showed the clear characteristic diffraction patterns formed by crystal planes, revealing a higher crystallinity of products in Figure 4f.

### 3.4. FT-IR Analyses

**Figure 5:**
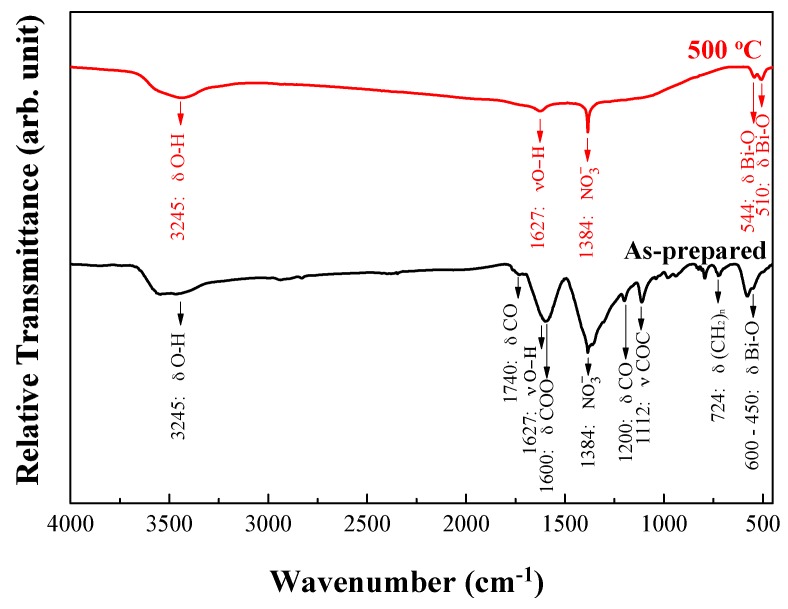
FT-IR spectrum of as-obtained and heat-treated Bi_2_O_3_ powders prepared using the sol-gel process.

FT-IR spectrometer was used to measure the bonding conditions of different Bi_2_O_3_ powders. In Figure 5, the as-prepared powders had an absorption peak at 450–600 cm^−1^, which is the stretching vibration absorption band caused by Bi-O bonding [43]. Sequentially, the vibration of (CH_2_)_n_, C-O and C-O-C groups contributed at 700–1000 and 1100–1200 cm^−1^. The peaks at 1285 and 1740 cm^−1^ resulted from the –COOH and C=O ester functional groups [44,45]. After heat-treatment at 500 °C, two characteristic peaks of *α*-Bi_2_O_3_ were detected at 510 and 544 cm^−1^ [46]. Three more absorption bands at 384, 1624, and 3425 cm^−1^ were, respectively, contributed by the NO_3_ group and O-H bonding in water molecules, suggesting the existence of NO_3_ and OH functional groups on the Bi_2_O_3_ surface [47]. The crystallinity phenomenon was similar to XRD and SAED results.

### 3.5. Characterization of Bi_2−x_Zr_x_O_3+x/2_ Powders

In the following, Bi_2−x_Zr_x_O_3+x/2_ powders were synthesized by adjusting x values from 0 to 1.0, in 0.2 steps. Through tuning the concentration of zirconyl nitrate hydrate precursors, the effects of the powders’ composition and the effect of annealing temperatures on various ratios were investigated as well.

### 3.6. XRD Analyses

**Figure 6:**
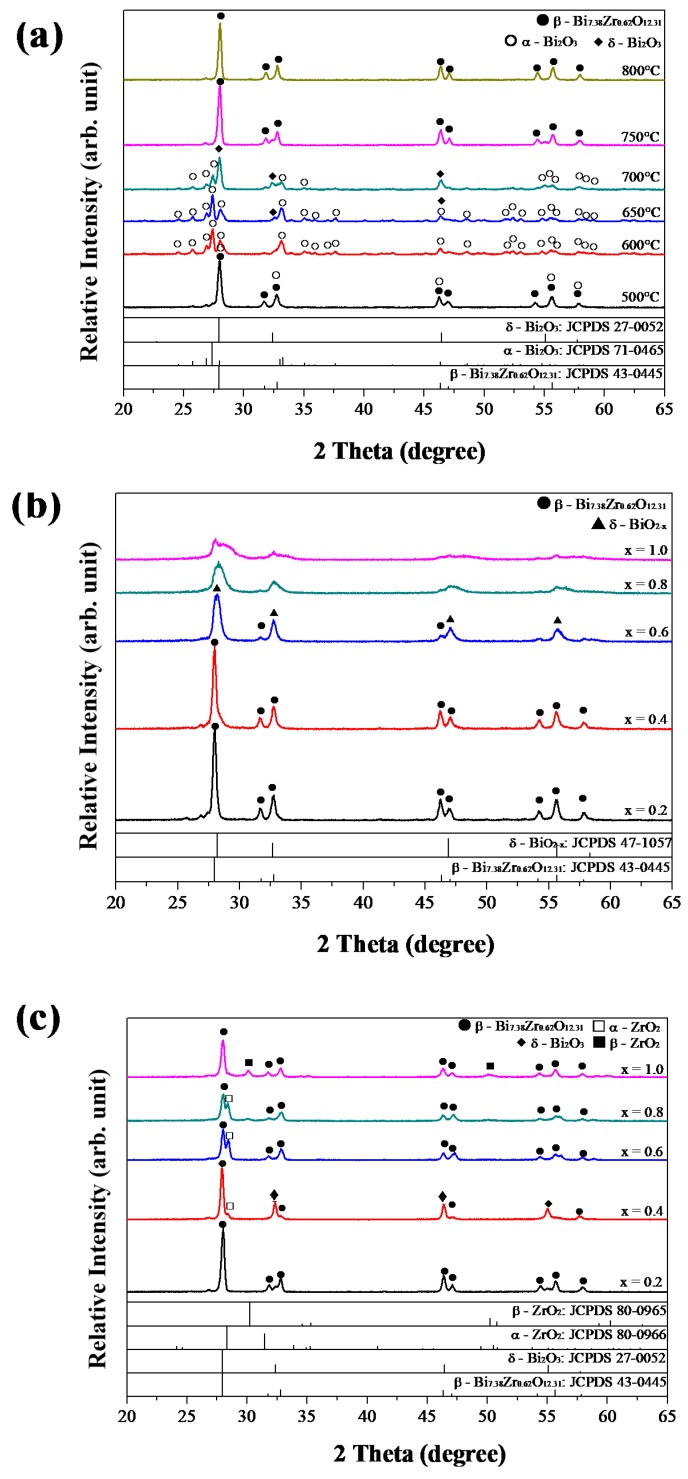
XRD patterns of (**a**) Bi_1.8_Zr_0.2_O_3.1_ after heat treatment at different temperatures and Bi_2−x_Zr_x_O_3+x/2_ (x = 0.2 to 1.0, 0.2 each) after heat treated at (**b**) 500 and (**c**) 750 °C.

For Bi_2−x_Zr_x_O_3+x/2_ systems, most studies adopted heating temperatures above 650 °C, so the synthesized Bi_2−x_Zr_x_O_3+x/2_ powders were baked at temperatures ranging from 500 to 800 °C and analyzed using X-ray diffraction. The results are shown in Figure 6. In Figure 6a, after heat treatment at 500 °C for 2 h, Bi_1.8_Zr_0.2_O_3.1_ powders mainly exhibited a tetragonal *β*-Bi_7.38_Zr_0.62_O_12.31_ phase with minor *α*-Bi_2_O_3_, which corresponds with the results reported by Sood et al. [48]. With the temperatures increasing, *α*-Bi_2_O_3_ phase became dominant until 700 °C. Over 750 °C, the high-temperature stable *β*-Bi_7.38_Zr_0.62_O_12.31_ phase appeared and was retained after cooling in the oven [49]. As for Bi_2−x_Zr_x_O_3+x/2_ powders, when x ≤ 0.4, the main structure was governed by *β*-Bi_7.38_Zr_0.62_O_12.31_ phase after being heated at 500 °C, as shown in Figure 6b. At x = 0.6, characteristic peaks right-shifted to the *δ*-BiO_2−x_ phase, and then the crystallinity decreased as x values increased. It was reported that metastable cubic ZrO_2_ phase transformed at 460–500 °C [50]. Therefore, a simultaneous phase transformation of Bi_2_O_3_ and ZrO_2_ would result in the solid solution effect influencing the crystallization reaction. XRD patterns of various Bi_2−x_Zr_x_O_3+x/2_ powders after being heated at 750 °C are further shown in Figure 6c. When x = 0.2, the major phase was the tetragonal *β*-Bi_7.38_Zr_0.62_O_12.31_ phase. As x increased to 0.4, a transient minor phase cubic *δ*-Bi_2_O_3_ phase appeared. From x values of 0.6–1.0, the tetragonal *β*-Bi_7.38_Zr_0.62_O_12.31_ phase dominant again, and the secondary phase became cubic ZrO_2_.

### 3.7. Morphology and Microstructure

**Figure 7:**
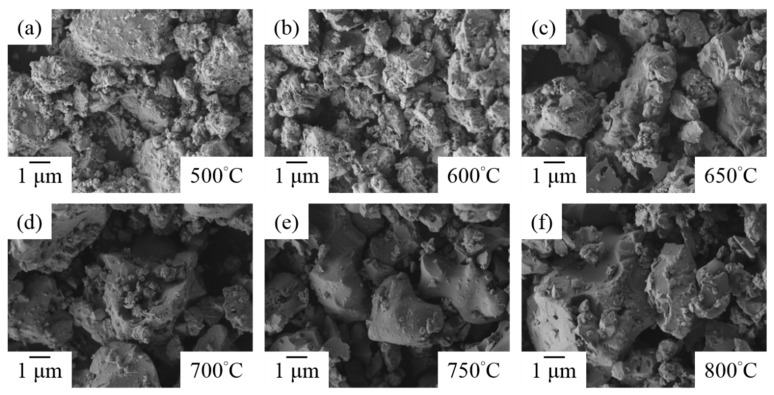
SEM images of Bi_1.8_Zr_0.2_O_3.1_ powders after heat treatment at temperatures of: (**a**) 500 °C; (**b**) 600 °C; (**c**) 650 °C; (**d**) 700 °C; (**e**) 750 °C; and (**f**) 800 °C.

**Figure 8:**
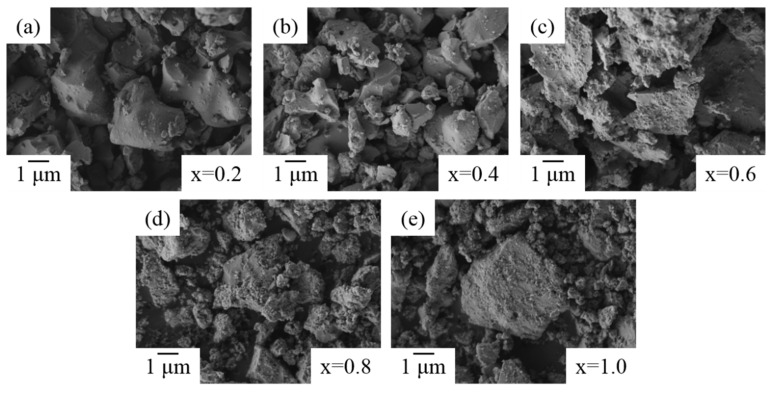
SEM images of Bi_2−x_Zr_x_O_3+x/2_ powders after 750 °C heat treatment at: (**a**) x = 0.2; (**b**) x = 0.4; (**c**) x = 0.6; (**d**) x = 0.8; and (**e**) x = 1.0.

To further evaluate the temperatures’ effects on the microstructure of ZrO_2_-doped Bi_2_O_3_ powders, SEM images of Bi_1.8_Zr_0.2_O_3.1_ powders treated at various temperatures are shown in Figure 7. Between 500 and 650 °C, the powders presented a rougher outline. Then, at ≥700 °C, the irregular particles became fewer due to the phase transformation of *δ*-Bi_2_O_3_, which contained 70–75% liquid phase sintering [44], thus the surface roughness or fine particles reduced with the temperature increase. However, as zirconium concentration increased, more fine particles were generated because of the precipitation of ZrO_2_, as shown in Figure 8a–e.

**Figure 9:**
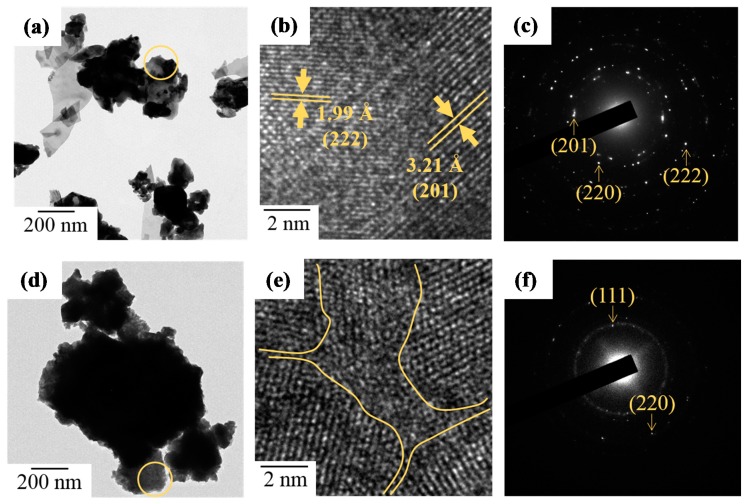
TEM images and SAED patterns of (**a**–**c**) Bi_1.8_Zr_0.2_O_3.1_ and (**d**–**f**) Bi_1.0_Zr_1.0_O_3.5_ powders after heat treatment at 500 °C.

**Figure 10:**
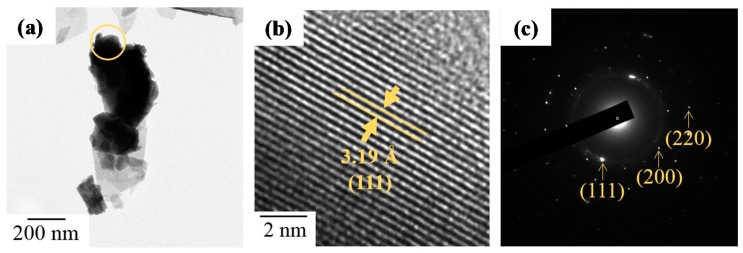
(**a**,**b**) TEM images and (**c**) SAED pattern of Bi_1.0_Zr_1.0_O_3.5_ powders after heat treatment at 750 °C.

In addition, TEM observations displayed a high crystallinity of heat treated ZrO_2_-doped Bi_2_O_3_ powders. In Figure 9, TEM images and SAED patterns of Bi_1.8_Zr_0.2_O_3.1_ and Bi_1.0_Zr_1.0_O_3.5_ powders after heat treatment at 500 °C are shown. At a low zirconium concentration, Figure 9a–c indicates that Bi_1.8_Zr_0.2_O_3.1_ powders are of a flake-stacking structure and have the characteristic d-spacing of 1.99 and 3.21 Å for (201) and (222) planes in the *β*-Bi_7.38_Zr_0.62_O_12.31_ phase. As the proportion continued to rise, the crystallinity of Bi_2_O_3_ became weak; a structure of short-term order was observed in Bi_1.0_Zr_1.0_O_3.5_ powders and its diffraction ring also became blurrier and wider, as shown in Figure 9e,f. After heat treatment at 750 °C, the appearance of Bi_1.0_Zr_1.0_O_3.5_ became sheet-like. According to the d-spacing and characteristic diffraction patterns in Figure 10, the obtained material is a mixture of *δ*-Bi_7.38_Zr_0.62_O_12.31_ and t-ZrO_2._

### 3.8. Radiopacity of Bi_2−x_Zr_x_O_3+x/2_ Powders

To evaluate the radiopacity properties of the products in this study, various Bi_2−x_Zr_x_O_3+x/2_ powders were made into specimens 10 mm in diameter and 1 mm in thickness. After being solidified, their related images were acquired through exposure to X-ray and the resulting images were examined according to the grey levels of aluminum standard.

**Figure 11:**
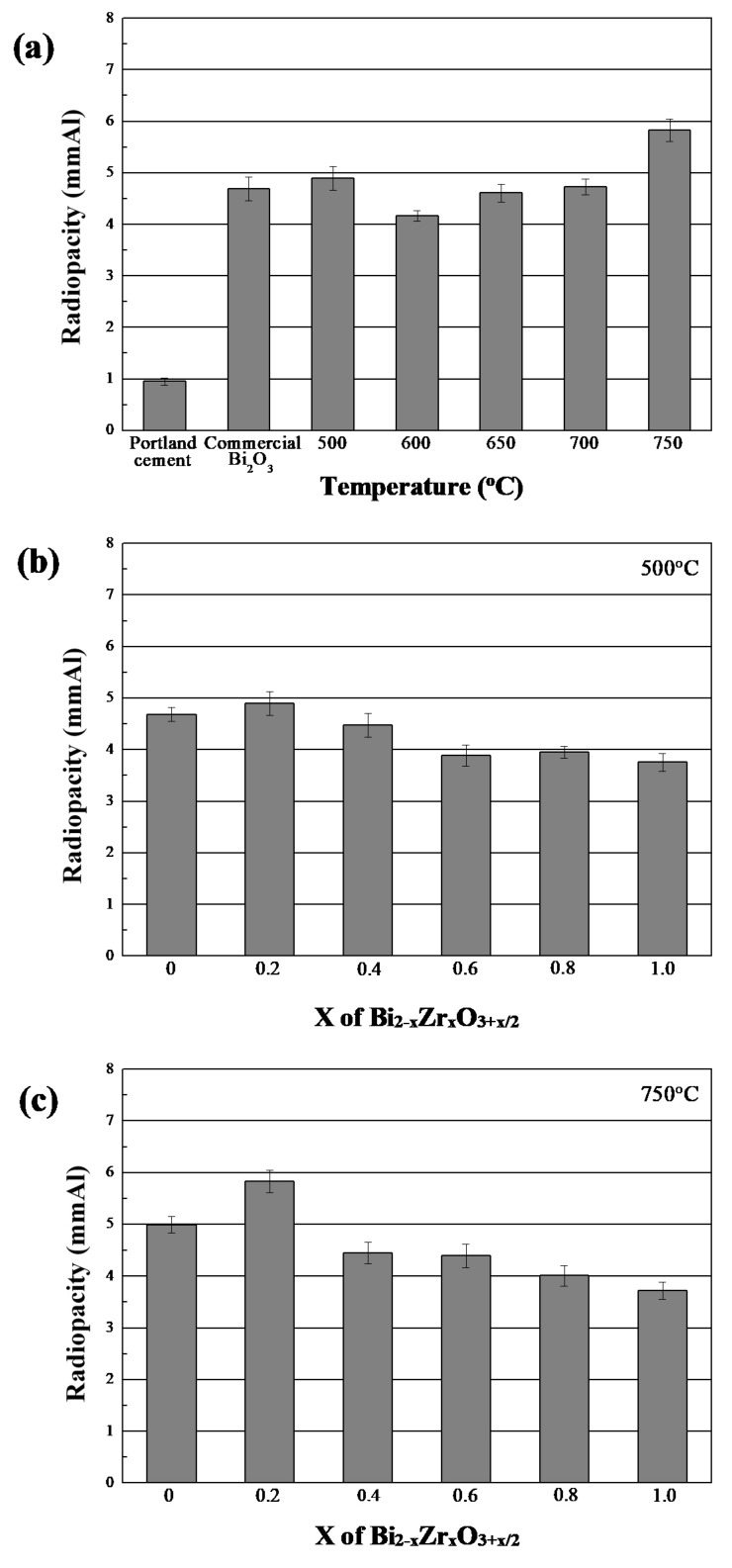
(**a**) Comparison of radiopacity of heat-treated Bi_1.8_Zr_0.2_O_3.1_ powders and commercial ceramic materials. Radiopacity of various Bi_2−x_Zr_x_O_3+x/2_ powders after heat treatment at: (**b**) 500 °C; and (**c**) 750 °C.

Based on previous results, it is noticeable that the crystallinity of heat-treated Bi_2−x_Zr_x_O_3+x/2_ powders was stable. Thus, two heating temperatures, 500 and 750 °C, were chosen to evaluate the composition effects on radiopacity properties. Figure 11a shows the radiopacity of commercial materials and heat-treated Bi_1.8_Zr_0.2_O_3.1_ powders. Originally, the radiopacity of pure Bi_2_O_3_ was higher than pure Portland cement and was evidently influenced by the phase composition. Below 750 °C, *α*-Bi_2_O_3_ phase was the dominant structure and had a nearly identical radiopacity of around 4.6 mmAl. As *β*-Bi_7.38_Zr_0.62_O_12.31_ appeared at 750 °C, a significant improvement of over 5.83 mmAl was measured. For the composition effects, whether at 500 or 750 °C, a higher zirconium proportion resulted in a lower radiopacity performance. In other words, bismuth oxide was still the ideal material with a relatively higher radiopacity compared to zirconium oxide. By combining the complete radiopacity results in Table 1, it is clear that most materials improved in radiopacity properties after a 750 °C heat treatment. When x was 0.2, heat-treated ZrO_2_-doped Bi_2_O_3_ exhibited the highest radiopacity (5.83 ± 0.22 mmAl). However, the radiopacity reduced as x values increased, which might be attributed to the possibility that more zirconium ions could not provide an equal ability to block or absorb the transmission of X-ray, and also affected the dominant structure of obtained powders, shifting from *δ*-Bi_7.38_Zr_0.62_O_12.31_ to *δ*-Bi_2_O_3_.

## 4. Conclusions

In this study, the radiopacity properties of as-prepared and heat-treated ZrO_2_-doped Bi_2_O_3_ powders synthesized using sol-gel processes were investigated. Regarding microstructures, heat-treated powders were composed of *β*-Bi_7.38_Zr_0.62_O_12.31_ and *α*-Bi_2_O_3_ phases at temperatures below 700 °C, and governed by *β*-Bi_7.38_Zr_0.62_O_12.31_ phase beyond 750 °C. Furthermore, for a fixed x value, the roughness of powder surface becomes smoother as heating temperatures increase. As for radiopacity evaluation, the performances were generally enhanced after heat treatments, and also improved with increasing temperatures. When x value equaled to 0.2, the maximum radiopacity value measured after a 750 °C heat treatment was 5.83 ± 0.22 mmAl.

## Figures and Tables

**Table 1:** Radiopacity properties of sol-gel Bi_2–x_Zr_x_O_3+x/2_ powders.

Temperature (°C)	Means and Standard Deviations of Radiopacity * (mmAl)							
	PC	PC/Bi_2_O_3_	0	0.2	0.4	0.6	0.8	1.0
	0.96 ± 0.07	4.69 ± 0.23						
**500**			4.68 ± 0.13	4.90 ± 0.23	4.48 ± 0.23	3.89 ± 0.21	3.95 ± 0.21	3.75 ± 0.17
**600**				4.17 ± 0.10				
**650**				4.61 ± 0.17				
**700**				4.73 ± 0.15				
**750**			4.99 ± 0.16	5.83 ± 0.22	4.45 ± 0.20	4.39 ± 0.22	4.01 ± 0.20	3.72 ± 0.16

PC: Portland cement, Bi_2_O_3_: commercial bismuth oxide (AVROS). * bold numbers indicate the x values of Bi_2–x_Zr_x_O_3+x/2_ powder prepared by sol-gel process.
